# Case of Abscess of the Left Lung; Thickening and Partial Ulceration of the Pericardium; Adhesion of the Right Lung, and Extensive Tubercular Deposition through the Entire Pulmonary Structure; with Obscure Diagnosis

**Published:** 1846-03

**Authors:** A. B. Smith

**Affiliations:** Ludlowville, N. Y.


***Case of Abscess of the Left Lung; thickening and partial Ulceration of the Pericardium; Adhesion of the Right Lung, and extensive Tubercular Deposition through the entire Pulmonary Structure; with Obscure Diagnosis.*** By A. B. Smith, M. D., of Ludlowville, N. Y.

Mr. Editor,—A very interesting article lately appeared in your Journal, by Dr. Swett, of New York, illustrative of the difficulty of correct diagnosis in some cases of pulmonary and cardiac affections. Should the following case appear to you to possess sufficient interest, in connection with the same subject, to justify its publication, it is at your disposal.

Campfield Tompkins, æt. 51, was a farmer of good health until about three years since, when, as the family believe, he received an injury by a fall from the scaffolding in a barn.

From that time until April, 1844, the period of his attack with the illness of which he died, he had occasionally, on rising in the morning, attacks of quite severe pain in the left hypochondrium. It, however, lasted but a short time, and left him feeling nearly as well as before, not interfering at all with the business of his vocation.

At the time above noted, while working in the field, he was attacked with severe pain in the left hypochondrium (that being the place to which he always pointed as the seat of most of his pain,) nausea, pain in the head and fever. Had a very restless night, was considerably worse in the morning. He however attempted to labor again, thinking thereby to get rid of his bad feelings, but he was soon obliged to desist, the symptoms becoming aggravated. Took a cathartic of sulph. magnesia which operated pretty freely, but procured no relief. The following day he called a physician, who gave a cathartic followed with a relaxing diaphoretic; and left orders for the application of a sinapism over the seat of the pain. This removed the most urgent symptoms, but he was by no means convalescent, but from that time until his death, continued, with only transient periods of apparent convalescence, to decline.

From this time until March, 1845, when I first saw him, he had most of the time been under an alternative, antiphlogistic course of treatment—and often with considerable relief; worn tart. emetic pustulating plasters over the seat of the pain; complained of a tightness and fulness under the right breast, which soon succeeded his first attack, and continued to harass him the remainder of his life.

When I first saw him, he had the following symptoms:—a sallow, rather sunken countenance, tongue slightly coated with a yellow fur; pulse a little accelerated; skin preternaturally warm and dry; pain in the left hypochondrium; tightness and fulness under the right breast; bowels costive; urine scanty and high colored; also at times a slight hacking cough, attended with scanty mucous expectoration. Gave a cathartic, to be followed with blue pill every night, if the bowels did not remain sufficiently soluble, and a solution of tart. emet. and ipecac.; the latter to be discontinued when the febrile action subsided. Saw him three days after: sallowness mostly gone, tongue cleaning; no cough; tightness and fulness less frequent and distressing, and bowels regular. He sits up and walks about most of the time.

Prepared a decoction of *polygala senega*, *taraxacum*, *ipecac.* and *rhubarb*, which had no visible effect; did not supersede the use of the laxative pills, which we designed it should do. Discontinued this; prescribed *tinct. iodine*, to be taken and applied externally over the seat of the pain, and a pill of *mass. hydrarg.*, *ipecac.* and *ext. taraxacum*:—to be taken every night on going to bed. Under this he appeared to improve, until he caught cold, when we had to discontinue the internal use of iodine, as it disagreed with his stomach and created a fever.

Until a short time previous to this, we had supposed the case to be one of impairment chiefly of the nutritive function, from a general glandular disease, in which the lungs were not seriously implicated, believing their morbid symptoms to be mostly sympathetic, from their mild and intermittent character. But we had to change our opinion somewhat by a closer examination, which revealed to us the following symptoms:—Countenance sunken; pulse sixty and quite feeble; tongue slightly coated with a white fur; slight hacking cough on rising in the morning, with but little and at times no expectoration; tightness and fulness periodical, occurring about half past one P.M., and remaining until about three—attended with some fever; pain in the left hypochondrium, dull and aching, with complete intermissions; but soreness constant and at times exquisite.

The right side of the chest was considerably enlarged, the ribs projecting about twelve lines in front of the sternum, very resonant, with a preternaturally strong respiratory murmur, though otherwise healthy. The left side was much contracted, had no motion on respiration, elicited a dull sound on percussion, and could detect *no* respiratory murmur on the left of the mediastinum. From this we concluded that the left lung was totally impermeable to air, although we could not satisfy ourselves as to the cause. The heart's action was healthy as far as we could determine.

Some three days after this, the patient began more rapidly to decline—was very restless, though in but little pain; most of the time none at all—manifested nothing new, except that the tongue assumed the raw-beef appearance.

Being now certain that no medicine could reach his case, our treatment from that time was only palliative. With a little morphine, &c., he was quite comfortable, and rested pretty well at night. Had no hectic, unless the slight fever attending, or causing the tightness and fulness, might be so called. Had no night sweats or any other colliquative discharge, but continued gradually, and at times almost imperceptibly, to waste away, until the last sands of life had ebbed out.

*Autopsy twenty-four hours after death:*—Emaciation extreme; no discoloration, or tympanitis.

After opening the chest in the usual way, I commenced the elevation from the right lateral inferior part of the sternum and cartilages, with the following appearances, viz.:—Under the right breast, as low down as the fifth rib, commencing on the cartilage, and extending laterally, was an adhesion of the lung for about thirty lines; which, in addition to the pleura for its bond, had several cartilaginous bands. There were also several incipient tubercles in the inferior margin of the anterior lobe. With these exceptions, the right lung appeared perfectly healthy, had no tubercles, miliary or otherwise, in the summit. I then divided the diaphragm to the left lateral incision, without, however, opening into the left pleural cavity, as it was firmly adherent to the pericardium: the diaphragm was also much thickened. On extending the incision upwards through the mediastinum I found that much thickened, and also firmly adherent to the pericardium; and that again to the costal pleura, through the whole extent of its contact, except where extending ulceration had burrowed in. Through these openings, yellow and not very fœtid pus issued quite freely. Turning the sternum back, we found the left pleural cavity completely occupied with pus, the lung being entirely disorganized, and the bronchi obliterated. We also discovered near the ensiform cartilage, at the right margin of the sternum, a large tubercle imbedded in the pleura in the caseous stage, and two near the superior part of the sternum; one of which was quite small, situated upon the first bone of the sternum, and the other a very large one, being over twelve lines in its lateral diameter, upon the cartilage of the first rib. The *heart* was of the usual size and healthy. Being obliged to hasten the autopsy we made no further examination of the chest.

The stomach and bowels, with their appendages, were healthy, except the mesenteric glands, which had quite large patches of miliary tubercles, with the interspaces healthy. The liver and spleen also contained the caseous tubercle, with, however, no other appearance of disease. In the appearance of the latter we were much disappointed, expecting from the symptoms, evidence of severe disease, the left hypochondrium having been the seat of most of the pain of which he complained. But doubtless this was sympathetic.

The singularity and much of the interest of this case consist in the absence of many, I might say most of the symptoms attendant on such a pathological condition, and the mild and equivocal character of those present. As far as I can learn, he never complained of pain or soreness in the chest (and with a view to detect the latter I have often thoroughly examined him,) had no dyspnœa; and never a harassing cough, and but little expectoration; slept perfectly quiet on either side; had no rigors,—or, as I conceive, hectic proper, and no night sweats. The character of the symptoms we will not again enumerate, as they have been sufficiently described.

Now in the light of these facts, the inquiry suggested to my mind, and one that I have not been able yet to satisfy is,—how could inflammation, sufficiently active as to cause hepatization and softening, have thus occurred in this occult manner and without producing more strongly marked symptoms?

I am sure that had there been an ordinary susceptibility of the system, disease could not have effected such extensive ravages, upon organs playing so important a part in the animal economy, and so essential to life, without having caused the most exquisite suffering, and giving evidence by unequivocal signs and symptoms of the character and extent of the disease. I question seriously whether such extensive disorganization could have thus occurred in any other than a system so devoid of sensibllity.—*New York Journal of Mediciue.*

